# Burden and contributing factors to overweight and obesity in young adolescents in Addis Ababa, Ethiopia

**DOI:** 10.1111/mcn.13479

**Published:** 2023-04-04

**Authors:** Roisin E. Drysdale, Amare W. Tadesse, Alemayehu Worku, Hanna Y. Berhane, Sachin Shinde, Isabel Madzorera, Deepika Sharma, Wafaie W. Fawzi

**Affiliations:** ^1^ DSI‐NRF Centre of Excellence in Human Development University of the Witwatersrand Johannesburg South Africa; ^2^ Addis Continental Institute of Public Health Addis Ababa Ethiopia; ^3^ Department of Infectious Disease Epidemiology London School of Hygiene and Tropical Medicine London United Kingdom; ^4^ School of Public Health Addis Ababa University Addis Ababa Ethiopia; ^5^ Department of Global Health and Population, T. H. Chan School of Public Health Harvard University Cambridge Massachusetts USA; ^6^ Center for Inquiry into Mental Health Pune India; ^7^ Global Nutrition Cluster, UNICEF New York City New York USA; ^8^ Department of Epidemiology, T. H. Chan School of Public Health Harvard University Cambridge Massachusetts USA; ^9^ Department of Nutrition, T. H. Chan School of Public Health Harvard University Cambridge Massachusetts USA

**Keywords:** adolescent, adolescent nutrition, low‐income countries, nutritional status, obesity, overweight

## Abstract

The prevalence of overweight/obesity in adolescents has increased globally, including in low‐ and middle‐income countries. Early adolescence provides an opportunity to develop and encourage positive health and behavioural practices, yet it is an understudied age group with limited information to guide and inform appropriate interventions. This study aims to determine the prevalence of overweight/obesity in young adolescents, aged between 10 and 14 years attending public schools in Addis Ababa, Ethiopia, and to explore the contributing factors. A cross‐sectional school‐based study was conducted. Adolescents completed individual questionnaires. Weight (kg) and height (m) measurements were converted to BMI‐for‐age and gender *z*‐scores. Multivariate regression analysis was conducted to determine the associated factors. The overall prevalence of overweight/obesity was 8% among adolescents aged 10–14 years and it was significantly higher in females (13%) than males (2%). The diet quality for the majority of the adolescents was inadequate, putting them at risk for poor health outcomes. The contributors to overweight/obesity were different between males and females. Age and no access to a flush toilet were negatively associated with overweight/obesity in males and access to a computer, laptop or tablet was positively associated. In females, menarche was positively associated with overweight/obesity. Living with only their mother or another female adult and an increase in physical activity were negatively associated with overweight/obesity. There is a need to improve the diet quality of young adolescents in Ethiopia and understand the reasons why females are less physically active to limit the risk of poor diet‐related health outcomes.

## Key messages

- Prevalence of overweight/obesity in young Ethiopian adolescents was 8% with a significantly higher rate in females (13%) than in males (2%).
- Despite no association between diet and overweight/obesity, the diet quality for the majority was inadequate putting them at risk for poor health outcomes.
- Many of the contributors to overweight/obesity were biological (menarche, age) or related to the household (parent lived with), factors individual adolescents cannot control, unlike physical activity or diet.
- Physical activity was negatively associated with overweight/obesity in females who were less active than males. Understanding reasons and barriers to physical activity may assist in reducing overweight/obesity prevalence.

## 1 INTRODUCTION

The prevalence of overweight and obesity has increased dramatically since the 1980s with nearly a third of the global population now classified as overweight or obese (The GBD 2015, Obesity Collaborators, 2017). While the burden is mainly in high‐income countries, low‐ and middle‐income countries are not exempt from this growing phenomenon (Global Nutrition Report, 2020) with sub‐Saharan Africa (SSA) in particular, now battling the double burden of malnutrition where both under‐ and over‐nutrition occur simultaneously, coupled with micronutrient deficiencies and diet‐related diseases such as diabetes (Mbogori & Mucherah, 2019). This is said to be driven by a nutrition transition where communities move away from their traditional diets of staple starchy foods to diets with calorie‐dense foods that have limited nutritional value (Mbogori & Mucherah, 2019; Popkin, 2004; Watson et al., 2021). At the same time, an economic transition is taking place in several countries where the duration and intensity of physical activity decrease as socioeconomic status increases (Steyn & Mchiza, 2014).

Ethiopia, like many other lower‐ and middle‐income countries, is facing the nutrition transition and in recent years, has seen an increase in the prevalence of overweight/obesity in both adults and children (Global Nutrition Report, 2020; INDEX Project, 2018). Between 2000 and 2016, the prevalence of overweight/obesity in adults increased from 32% to over 50% and at 35%, women continue to experience higher rates than men (Global Nutrition Report, 2022). With the increasing obesity rates, the country is also seeing an increase in diabetes and other diet‐related noncommunicable diseases in adults over the age of 18 years and is currently not on track to meet any of the 2025 Global nutrition targets (Global Nutrition Report, 2020). Similar trends are being seen for children and adolescents aged between 5 and 19 years. Between 2000 and 2016, the prevalence of males overweight/obese in Ethiopia increased from 2% to 5%. For females, this was much higher, and the prevalence increased from 5% to 14% (Global Nutrition Report, 2022). For adolescents, those aged between 10 and 19 years, the rate of increase has been much higher with studies indicating prevalence rates of <10% in 2014/2015 compared with rates of >25% in 2021/2022 (Belay et al., 2022; Biadgilign et al., 2021).

Adolescence is a time were many physical and emotional changes take place. Early adolescence, between 10 and 14 years, in particular, is often when individuals begin going through the physiological change of puberty (Farello et al., 2019; Perry, 2013). The changes in body composition experienced during puberty often go on to influence body characteristics and experiences in adulthood (Adami et al., 2020; Farello et al., 2019; Rogol et al., 2002). This is also a time when individual health attitudes, behaviours and gender norms are shaped (Jacob et al., 2017; Jones et al., 2019; Kågesten et al., 2016) and have been shown to be an opportune time to develop and encourage positive health and behavioural practices (Blum et al., 2014; Patton et al., 2016). This is because those picked up during this time are likely to be maintained into adulthood (Frech, 2012; Lau et al., 1990; Telama et al., 1997). In addition, adolescents who are overweight or obese are more likely to remain so throughout adulthood and are prone to suffer from diet‐related chronic diseases (Dehghan et al., 2005; Sahoo et al., 2015), cardiovascular diseases (Herouvi et al., 2013; Sahoo et al., 2015; Sommer & Twig, 2018) and may experience psychosocial consequences (Chu et al., 2019; Sagar & Gupta, 2018). Targeting this age group with healthy living education or interventions may reduce or slow the current overweight/obesity epidemic (Haire‐Joshu & Tabak, 2016). However, it is an understudied age group and there is limited evidence to guide and inform appropriate interventions (Blum et al., 2014; Lane et al., 2017). We aimed to determine the prevalence of overweight/obesity in young adolescents, aged between 10 and 14 years attending public schools in Addis Ababa, Ethiopia, and to explore the contributing and associated factors. Jones et al. (2019) highlight that socio‐cultural gender norms are enforced and consequential in early adolescence and will set different trajectories as males and females transition to adulthood. Males and females also experience puberty differently and have different needs throughout adolescence and thus, we conduct our analysis on each gender separately.

## 2 METHODS

A school‐based cross‐sectional study was conducted through November 2019 in government primary schools in the 10 subcities of the Ethiopian capital city, Addis Ababa. Multistage random sampling was used to select the schools and students to participate in the study. Two government primary schools were randomly selected from each of the 10 subcities. Only adolescents aged between 10 and 14 years and enroled in Grades 5–8 were invited to participate. Using the school registration lists for the current academic year, 15 students from each of the 4 grades were randomly selected. In total, 60 students, both male and female, from each of the 20 schools were enroled, making a total of 1200 adolescents. Gender was not considered in the sampling strategy.

Data were collected through an interviewer‐administered questionnaire that captured information on demographic characteristics, socioeconomic status, food insecurity, access to technology or media, physical activity and dietary practices. Anthropometric measures were taken from each participant, including weight (kg) and height (m), using digital scales and stadiometers.

Data were captured and analyzed using Stata Version 14 (StataCorp. LLC). An asset index was calculated using principal component analysis based on ownership of the following assets: electricity, radio, television, mobile phone, refrigerator, washing machine, computer, camera, DVD or CD player, bed or mattress, table, chair, cabinet or cupboard, bicycle, motorcycle, car and solar panel. The asset index was divided into tertiles. Participants were recorded as being food insecure if in the last 30 days they reported there being no food in the house, someone in the household going to bed hungry, or spending a whole day and night without eating. Diet quality was measured using the Global Diet Quality Score (GDQS) which classifies 25 food groups into healthy, unhealthy and unhealthy in excess as per the Center for Dietary Assessment guidelines (Intake, 2021). Students were asked to report how frequently they ate the 25 groups in the previous 7 days and were given a score that ranged between 0 and 49. A score ≥ 23 is associated with a low risk of nutrient inadequacy and related dietary outcomes, a score ≥15 and <23 indicates moderate risk, while a score <15 indicates high risk (Intake, 2021). Access to media or technology was measured through the adolescent's access to a cell phone (their own or someone else's), access to a laptop, computer, or tablet, access to the internet and access to social media. These were recorded as binary, yes or no, variables. Physical activity was measured through the number of days in the week individuals were physically active for at least an hour and was recorded as none, between 1 and 3 days or between 4 and 7 days.

Body mass index (BMI) was calculated by weight (kg) divided by height (m) squared. BMI‐for‐age and gender *z*‐scores were calculated based on the WHO growth reference guidelines (World Health Organization, 2021a). Being overweight was defined as a BMI‐for‐age *z*‐score ≥1 and <2. Obese was defined as a BMI‐for‐age *z*‐score ≥2 (World Health Organization, 2021a). Overweight and obese were combined into one category due to small numbers.

2.1

Categorical descriptive statistics are presented as proportions and frequencies. Continuous descriptive statistics are presented as mean, and standard deviations. Pearson's *χ* ^2^ was used to compare differences in sociodemographic variables and potential contributors between males and females. Univariate logistic regression was employed to determine the characteristics of adolescent overweight/obesity on all possible contributing variables. Potential contributors were chosen based on the conceptual framework of nutritional status among adolescents presented by Maehara et al. (2019). We considered individual (age, gender, menarche), household (sanitation, food insecurity) and parental (education, employment) factors as well as variables on behaviour (diet, physical activity) and access to technology or media (internet, social media, computer access). All regression analyses were carried out separately for males and females. Some variables were omitted in the univariate analysis for males due to collinearity, and include, but are not limited to, the experience of food insecurity, physical education classes and consumption of baked goods. Predictors with a *p* < 0.2 were included in the multivariate model. For males, these included age, paternal employment, toilet access and computer/laptop/tablet access. For females, they included who they lived with, maternal employment, toilet access, menarche, physical activity, computer/laptop/tablet access, number of meals eaten the previous day and consumption of fried food and processed meat. All statistical tests were considered significant with a *p* < 0.05. Regression analyses are presented as either odds ratio (OR) or adjusted odds ratio (AOR) and with 95% confidence levels. All models were adjusted for school clustering.

### 2.2 Ethical statement

Ethical approval for the study was granted by the Institutional Ethical Review Board of the Addis Continental Institute of Public Health (ACIPH/IRB/002/2019) and the Institutional Review Board of the Harvard T.H. Chan School of Public Health (IRB19‐0822). Parents of sampled adolescents were provided with an information sheet and requested to sign an informed consent form. If approved, the adolescents were provided with an information sheet and requested to sign an informed assent sheet. All information was kept confidential and no identifying information was recorded.

## 3 RESULTS

Table 1 shows the sociodemographic characteristics of the participants. Females made up a slightly higher proportion of the participants at 55% and the mean age was 13 (1.17) years for both males and females. A higher percentage of males (67%) stated that they lived with either both their parents or both a male and female adult compared with 55% of females. Nearly 40% of females lived with a female adult only compared with 27% of males. The difference in who the participants lived with was statistically significant (*x* ^2^ = 22.10, *p* < 0.001). A slightly higher proportion of males (67%) reported that their father was employed compared with 55% of females. There was a significant difference in the proportion of females either not knowing or living with their father or a male adult at 42% compared with 29% of males (*x* ^2^ = 21.21, *p* < 0.001). The majority of the participants (96%) had access to piped drinking water, either to their home or the neighbourhood and a high proportion did not have access to a flush toilet (males 81%; females 71%). The difference in toilet access between genders was statistically significant (*x* ^2^ = 13.87, *p* < 0.001).

**Table 1:** Sociodemographic characteristics of the participants

	Overall, *n* (%)	Males, *n* (%)	Females, *n* (%)
Sample size	1200 (100.0)	543 (45.25)	657 (54.75)
Age (years)			
10	54 (4.50)	24 (4.42)	30 (4.57)
11	188 (15.67)	98 (18.05)	90 (13.70)
12	294 (24.50)	133 (24.49)	161 (24.51)
13	344 (28.67)	137 (25.23)	207 (31.51)
14	320 (26.67)	151 (27.81)	169 (25.72)
Parents alive			
Both alive	1027 (85.58)	472 (86.92)	555 (84.47)
One deceased	162 (13.50)	66 (12.15)	96 (13.61)
Both deceased/don't know	11 (0.92)	5 (0.92)	6 (0.91)
Currently living with***			
Both parents/male AND female adult	724 (60.33)	362 (66.67)	362 (55.10)
Mother/female adult only	398 (33.17)	147 (27.07)	251 (38.20)
Father/male adult only	60 (5.00)	30 (5.52)	30 (4.57)
Siblings or no adult	18 (1.50)	4 (0.74)	14 (2.13)
Mother employed			
Yes	651 (54.25)	289 (53.22)	362 (55.10)
No	436 (36.33)	209 (38.49)	227 (34.55)
Don't know/no mother	113 (9.42)	45 (8.29)	68 (10.35)
Father employed***			
Yes	723 (60.25)	362 (66.67)	361 (54.95)
No	46 (3.83)	24 (4.42)	22 (3.35)
Don't know/no mother	431 (35.92)	157 (28.91)	274 (41.70)
Mother education			
None	225 (18.75)	110 (20.26)	115 (17.50)
Primary	430 (35.83)	188 (34.62)	242 (36.83)
Secondary	251 (20.92)	121 (22.28)	130 (19.79)
Higher education	67 (5.58)	24 (4.42)	43 (6.54)
Don't know/no mother	227 (18.92)	100 (18.42)	127 (19.33)
Father education***			
None	62 (5.17)	36 (6.63)	26 (3.96)
Primary	264 (22.00)	133 (24.49)	131 (19.94)
Secondary	237 (19.75)	117 (21.55)	120 (18.26)
Higher education	99 (8.25)	48 (8.84)	51 (7.76)
Don't know/No father	538 (44.83)	209 (38.49)	329 (50.08)
Drinking water access			
Piped to house	728 (60.67)	319 (58.75)	409 (62.25)
Piped to neighbourhood	420 (35.00)	201 (37.02)	219 (33.33)
Other	52 (4.33)	23 (4.24)	29 (4.41)
Toilet access***			
Flush toilet	293 (24.42)	105 (19.34)	188 (28.61)
Other (pit/latrine/bush/field/non)	907 (75.58)	438 (80.66)	469 (71.39)
Asset index			
Low	409 (34.08)	187 (34.44)	222 (33.79)
Mid	392 (32.67)	179 (32.97)	213 (32.42)
High	399 (33.25)	177 (32.60)	222 (33.79)

*Note*: *χ* ^2^ conducted to compare differences between gender.

* *p* < 0.05

** *p* < 0.01

*** *p* < 0.001.

In total, 8% of the participants were overweight/obese. The proportion of females overweight (13%) compared with males (2%) was significantly higher (*x* ^2^ = 46.67, *p* < 0.001). As shown in Table 2, the only variable relevant to just females was menarche with 44% reporting having started their menstrual periods. In terms of dietary factors, the mean GDQS score was similar for females and males at 20.2 (2.84) and 20.0 (2.61), respectively. Most males (86%) and females (80%) were at moderate risk for nutrient inadequacy and related poor health outcomes. However, females appeared to have better diets with a higher proportion being at low risk for nutrient inadequacy (*x* ^2^ = 7.29, *p* = 0.026). The majority of the participants (87%) reported having eaten three meals in the previous 24 h with little difference between males (88%) and females (85%). The mean number of unhealthy food groups consumed weekly for both males and females was 3 (0.03). When assessed by individual food groups, a higher proportion of females consumed processed meat weekly (*x* ^2^ = 9.14, *p* = 0.002). There were no significant differences in the remaining unhealthy food groups between gender. However, household food insecurity was higher in males (10%) than in females (6%) (*x* ^2^ = 6.74, *p* = 0.009). In terms of physical activity, only 14% of the participants were physically active for at least 1 h on 4 or more days a week. Females appeared to be less active than males with nearly half (48%) reporting no physical activity in a week compared with only 25% of males. This difference was statistically significant (*x* ^2^ = 71.57, *p* < 0.001). There were no significant differences in access to media or technology between males and females.

**Table 2:** Potential contributors to overweight/obesity

	Overall, *n* (%)	Male, *n* (%)	Female, *n* (%)		Overall, *n* (%)	Male, *n* (%)	Female, *n* (%)
*Dietary factors*							
Food insecure**				Eaten snacks in previous 24 h			
No	1111 (92.58)	491 (90.42)	620 (94.37)	No	567 (47.25)	268 (49.36)	299 (45.51)
Yes	89 (7.42)	52 (9.58)	37 (5.63)	Yes	633 (52.75)	275 (50.64)	358 (54.49)
Processed meat consumed weekly**				Sweets/ice cream consumed weekly			
No	690 (57.50)	338 (62.25)	352 (53.58)	No	409 (34.08)	186 (34.25)	223 (33.94)
Yes	510 (42.50)	205 (37.75)	305 (46.42)	Yes	791 (65.92)	357 (65.75)	434 (66.06)
Baked goods consumed weekly				Fried food consumed weekly			
No	25 (2.09)	7 (1.29)	18 (2.74)	No	548 (45.67)	235 (43.28)	313 (47.64)
Yes	1174 (97.91)	535 (98.71)	639 (97.26)	Yes	652 (54.33)	308 (56.72)	344 (52.36)
Risk of nutrient inadequacy and related outcomes*				Sweet drinks consumed weekly			
Low risk	179 (14.92)	65 (11.97)	114 (7.35)	No	594 (49.54)	287 (52.85)	307 (46.80)
Moderate risk	989 (82.42)	465 (85.64)	524 (79.76)	Yes	605 (50.46)	256 (47.15)	349 (53.20)
High risk	32 (2.67)	13 (2.39)	13 (2.89)				
*Female only*				*Physical activity*			
Menarche				Physically active for 1 h***			
Yes			289 (43.99)	None/don't know	456 (38.00)	138 (25.41)	318 (48.40)
No			56.01 (56.01)	1–3 days	576 (48.00)	301 (55.43)	275 (41.86)
				4–7 days	168 (14.00)	104 (19.15)	64 (9.74)
*Access to media/technology*							
Access to a cell phone				Access to social media			
Yes	956 (79.67)	427 (78.64)	529 (80.52)	Yes	76 (6.33)	35 (6.45)	41 (6.24)
No	244 (20.33)	116 (21.36)	128 (19.48)	No	1124 (93.67)	508 (93.55)	616 (93.76)
Access to a computer/laptop/tablet				Access to the internet			
Yes	174 (14.50)	79 (14.55)	95 (14.46)	Yes	42 (3.50)	21 (3.87)	21 (3.20)
No	1026 (85.50)	464 (85.45)	562 (85.54)	No	1158 (96.50)	522 (96.13)	636 (96.80)

*Note*: *χ* ^2^ conducted to compare differences between gender.

* *p* < 0.05

** *p* < 0.01

*** *p* < 0.001.

The results of the univariate analysis separated by gender are shown in Table 3. For males, only age (OR = 0.58, *p* = 0.036, 95% confidence interval [CI] = 0.35–0.97) and access to other toilet facilities such as a pit latrine, bush or no facilities (OR = 0.19, *p* = 0.007, 95% CI = 0.06–0.65) were negatively associated with being overweight/obese. For females, living with no adults or siblings only (OR = 2.97, *p* = 0.045, 95% CI = 1.02–8.64), menarche (OR = 2.47, *p* < 0.001, 95% CI = 1.70–3.61) and access to a computer or laptop at home (OR = 1.51, *p* = 0.033, 95% CI = 1.04–2.20) were found to be positively associated with being overweight/obese. Living with only their mother or another female adult was found to be negatively associated with overweight/obesity (OR = 0.57, *p* = 0.040, 95% CI = 0.33–0.97).

**Table 3:** Univariate analysis to determine contributors to overweight/obesity

	Male		Female			Male		Female	
	OR	95% CI	OR	95% CI		OR	95% CI	OR	95% CI
*Sociodemographic factors*									
Age	0.58*	0.35–0.97	1.06	0.85–1.33	Asset index	1.30	0.77–2.20	0.98	
Parents alive					Currently living with				
Both alive	Ref.		Ref.		Male and female adult	Ref.		Ref.	
One deceased	0.59	0.10–3.62	1.36	0.68–2.73	Mother/female guardian adult	1.24	0.30–5.17		0.33–0.97
Both deceased/don't know	1	–	1.36	0.15–12.63	Father/male guardian adult	1.53	0.26–8.98	0.57*	0.08–1.74
					Siblings or no adult	1	–	0.38	1.02–8.64
								2.97*	
Mother education					Father education				
None	Ref.		Ref.		None	Ref.		Ref.	
Primary	1.17	0.11–13.39	0.69	0.35–1.35	Primary	0.52	0.14–1.95	1.23	0.45–3.37
Secondary	2.77	0.25–30.80	0.71	0.33–1.54	Secondary	0.89	0.22–3.16	0.56	0.22–1.40
Higher education	1	–	1.16	0.53‐3.13	Higher education	1.47	0.17–12.92	1.18	0.40–3.45
Don't know/No mother	8.20	1.18–57.22	0.73	0.34–1.56	Don't know/no father	1		0.76	0.26–2.21
Mother employed					Father employed				
No	Ref.		Ref.		Yes	Ref.		Ref.	
Yes	0.87	0.26–2.84	1.78^a^	0.91–3.46	No	0.22^a^	0.04–1.25	1.76	0.41–7.59
Don't know/no mother	1.90	0.39–9.46	1.24	0.48–3.23	Don't know/no mother	0.29	0.04–1.96	1.32	0.25–7.04
Drinking water access					Toilet access				
Piped to house	Ref.		Ref.		Flush toilet	Ref.		Ref.	
Piped to neighbourhood	0.47	0.14–0.15	1.18	0.67–2.06	Other	0.19**	0.06–0.65	0.74^a^	0.51–1.08
Other	1		1.10	0.40–3.01					
*Dietary factors*									
Meals eaten in 24 h	0.55	0.20–1.53	1.56^a^	0.85–2.86	GDQS	1.05	0.78–1.41	1.03	0.92–1.15
No. of unhealthy food groups eaten on a weekly basis	0.96	0.65–1.42	1.06	0.90–1.23	Food insecure				
					No			Ref.	
					Yes			0.77	0.26–0.23
Eaten snacks in previous 24 h					Sweets and ice cream consumed weekly				
No	Ref.		Ref.		No	Ref.		Ref.	
Ye	0.83	0.29–2.38	1.05	0.77–1.46	Yes	0.83	0.17–4.09	0.79	0.53–1.18
Baked goods consumed weekly					Fried food consumed weekly				
No			Ref.		No	Ref.		Ref.	
Yes			2.68	0.32–22.15	Yes	0.65	0.16–2.61	1.30^a^	0.89–1.90
Sweet drinks consumed weekly					Processed meat consumed weekly				
No	Ref.		Ref.		No	Ref.		Ref.	
Yes	0.96	0.28–3.31	0.82	0.59–1.13	Yes	1.43	0.41–4.99	1.46^a^	0.96–2.20
*Female only*					*Physical activity*				
Menarche					Physically active for 1 h				
No			Ref.		None/don't know	Ref.		Ref.	
Yes			2.47***	1.70–3.61	1–3 days	0.57	0.16–2.05	0.64^a^	0.39–1.04
					4–7 days	1.34	0.19–9.49	1.30	0.62–2.72
*Access to media/technology*									
Access to a cell phone					Access to the internet				
No	Ref.		Ref.		No			Ref.	
Yes	0.90	0.28–2.91	1.32	0.69–2.56	Yes			0.32	0.04–2.44
Access to a computer/laptop/tablet					Access to social media				
No	Ref.		Ref.		No			Ref.	0.69–3.80
Yes	2.70^a^	0.82–8.92	1.51**	1.04–2.20	Yes			1.62	

*Note*: All models are adjusted for the school‐level clustering.

Abbreviation: CI, confidence interval.

^a^ *p* < 0.200.

* *p* < 0.05

** *p* < 0.01

*** *p* < 0.000.

The results of the multivariate analysis, carried out individually by gender, are shown in Table 4. For males, age (AOR = 0.55, *p* = 0.034, 95% CI = 0.32–0.96) and access to other toilet facilities (AOR = 0.21, *p* = 0.017, 95% CI = 0.06–0.76) remain negatively associated with being overweight/obese. Access to a computer, laptop or tablet at home was found to be positively associated with overweight/obesity in males (AOR = 3.01, *p* = 0.016 95% CI = 1.23–7.34). For females, menarche (AOR = 3.26, *p* < 0.001, 95% CI = 2.12–4.99) remained positively associated with overweight/obesity. Living with only their mother or another female adult was negatively associated with overweight/obesity (AOR = 0.50, *p* = 0.032, 95% CI = 0.26–0.94). Females who were physically active for at least an hour between 1 and 3 days were less likely to be overweight/obese than those who were physically inactive (AOR = 0.52, *p* = 0.019, 95% CI = 0.31–0.90), although more days of physical activity was not associated with the outcome. Access to a computer in the home was no longer found to be significantly overweight/obese in females.

**Table 4:** Multivariate analysis to determine contributors to overweight/obesity

	Male		Female	
	AOR	95% CI	AOR	95% CI
*Sociodemographic factors*				
Age (years)	0.55*	0.32–0.96		
Currently living with				
Both parents/male and female adult			Ref.	
Mother/female adult only			0.50*	0.26–0.94
Father/male adult only			0.98	0.26–3.71
Siblings or no adult			5.77	0.97–34.46
Mother employed				
No			Ref.	
Yes			1.88	0.87–4.07
Don't know/no mother			0.77	0.13–4.47
Father employed				
No	Ref.			
Yes	0.19	0.03–1.18		
Don't know/no mother	0.25	0.03–1.91		
Toilet access				
Flush toilet	Ref.		Ref.	
Other (pit/latrine/bush/field/non)	0.21*	0.06–0.78	0.75	0.44–1.26
*Dietary factors*				
Meals eaten in 24 h			1.61	0.84–3.07
Fried food consumed weekly				
No			Ref.	
Yes			1.29	0.80–2.11
Processed meat consumed weekly				
No			Ref.	
Yes			1.49	0.98–2.25
*Physical activity*				
Physically active for 1 h				
None/don't know			Ref.	
1–3 days			0.52*	0.31–0.90
4–7 days			1.29	0.54–3.10
*Access to media/technology*				
Access to a computer/laptop/tablet				
No	Ref.		Ref.	
Yes	3.01*	1.23–7.34	1.33	0.80–2.21
*Female only*				
Menarche				
No			Ref.	
Yes			3.26***	2.12–4.99

*Note*: All models are adjusted for the school‐level clustering.

Abbreviations: AOR, adjusted odds ratio; CI, confidence interval.

* *p* < 0.05

** *p* < 0.01

*** *p* < 0.000.

## 4 DISCUSSION

The findings from this study indicate that the prevalence of overweight/obesity in adolescents aged 10–14 years attending government primary schools in Addis Ababa is 8%, with the rate being significantly higher in females (13%) than males (2%). The results also highlight that factors associated with overweight/obesity vary between genders.

The significant difference in overweight/obesity between males and females is consistent with the global prevalence of overweight/obesity (World Health Organization, 2021b), as well as a number of studies conducted in SSA, including Ethiopia (Choukem et al., 2020; Danquah et al., 2020; Darling et al., 2020; Gali et al., 2017; Gebrie et al., 2018; Teshome et al., 2013). According to Hallam et al. (2016), intertwined biological, sociological and environmental factors likely account for gender differences in obesity. For example, males and females experience differences in their metabolic functions which contribute to differences in weight gain and loss (Shope, 2020). In addition, variations in hormones affect males and females differently with direct correlations on their metabolisms leading to changes in energy intake and expenditure (Hallam et al., 2016; Shope, 2020). Culturally, there is often a desire for women in SSA to be overweight (Choukem et al., 2020; Muthuri et al., 2014) and females are often expected to complete household chores leaving them with little time to be social or physically active (Adams et al., 2014; Makhata et al., 2021). While we did not compare household chores between the genders, we did find that females were less physically active than males and that higher levels of physical activity were negatively associated with overweight/obesity in females. To improve physical activity in young adolescent females, we need to first understand the reasons and any barriers they face and find ways to address them.

Although the prevalence of overweight/obesity was very low for males in this study, the associated factors were age, access to a computer and toilet access. As previously stated, increasing age is associated with higher overweight/obesity rates in previous studies conducted in SSA (Danquah et al., 2020; Darling et al., 2020). However, we found that as males got older, they were less likely to be overweight or obese. It is possible that in previous studies, a pooled gender analysis was conducted which affected the results. Males experience biological changes during puberty with increasing levels of testosterone leading to higher bone and muscle mass coupled with a loss of fat (Tanner, 1989). Based on this, we might expect that as males get older, they are less likely to be overweight or obese. Access to a computer, laptop or tablet was positively associated with overweight/obesity in male adolescents. It is possible that this association was due to increased screen time and low activity levels, both of which have been found to be associated with an increase in overweight/obesity (Adom et al., 2019; Fang et al., 2019; Sorrie et al., 2017; Teshome et al., 2013; Tripathi & Mishra, 2019). This is a common phenomenon across the globe with improved access to technology in low‐ and middle‐income counties creating a shift in physical activity levels, not only in adolescents but children and adults too (World Health Organization, 2018). Both male and female adolescents should be encouraged to maintain a healthy active lifestyle in spite of increased screen time. Finally, poor sanitation, in the way of access to a pit, chemical or no toilet, was negatively associated with overweight/obesity. While we did not measure infection, poor sanitation can lead to bacterial infections (Freeman et al., 2017) which in the long‐term increase the risk of undernutrition (Freeman et al., 2017; Hutton & Chase, 2017). On the other hand, access to poor sanitation may be an indication of low socioeconomic status and poverty (Akpakli et al., 2018; Angoua et al., 2018; Njuguna, 2019; Rhodes & McKenzie, 2018), a commonly reported contributor to undernutrition in low‐ and middle‐income countries (Ekholuenetale et al., 2020; Madjdian et al., 2018).

For females, menarche was positively associated with being overweight or obese, with those who had started menstruation being more than twice as likely to be overweight or obese. Previous studies have found similar associations and suggest that a higher BMI or excess weight leads to a lower menarche age (Baker, 1985; Barros et al., 2019; Biro et al., 2018; Mohamad et al., 2013) with malnutrition delaying the onset of puberty (Baker, 1985; Burt Solorzano & McCartney, 2010). While we did not measure the age of the first period, it is likely that the association found between overweight and menarche is similar to that found in previous studies. When assessing nutritional status in young adolescent females, future research should take into account the age of menarche as it is likely to be a confounding factor. Female adolescents who lived with only their mother or another female adult, compared with those who lived with both parents or both a male and female adult, were less likely to be overweight/obese. Current literature suggests that female‐headed households tend to be poorer (Milazzo & van de Walle, 2015) which as previously discussed, is commonly associated with undernutrition. However, in urban Ethiopia, there is only a 2% difference in poverty rates between male and female‐headed households (World Bank, 2020). Single motherhood on the other hand is a risk factor for poor child nutritional status (Ntoimo & Odimegwu, 2014) and those that live in dual‐parent homes tend to have better health outcomes (Lut et al., 2021; Scharte et al., 2012).

A number of the variables associated with overweight/obesity in both males and females were either biological (age, menarche) or related to the household (toilet access, who they live with), things individual adolescents cannot change. Variables an individual may have control over are physical activity and diet. With a mean score of 20 in the GDQS, as per the GDQS guidelines cut‐offs, the diet quality for the majority of the adolescents in this study puts them at moderate risk for poor diet quality outcomes and nutrient inadequacy (Intake, 2021). In addition, out of the five unhealthy food groups, the majority reported consuming three on a weekly basis with baked goods and sweets or ice cream among the most commonly consumed. We did not find an association between any dietary factors and overweight/obesity in this study, potentially due to how diets were measured. We only recorded the weekly consumption of food groups and were not able to measure the quantity or quality of the food eaten. We were also not able to consider individual factors, such as metabolism or energy outputs. While the GDQS has been validated for use in adolescents to evaluate nutrient adequacy (World Health Organization, 2021c), an analysis that takes into account overall calorie input and energy output should be considered for future research. That said, the results of the study do highlight that there is a need to improve the diets of young adolescents in Ethiopia to ensure they are not at risk of both under‐ and over‐nutrition, as well as diet‐related noncommunicable diseases such as micronutrient deficiencies and diabetes.

## 5 STUDY STRENGTHS AND LIMITATIONS

There is limited research that focuses on the burden of overweight/obesity in early adolescence, particularly those in Ethiopia. To our knowledge, this is also one of the few that has taken a gendered approach and thus, we have highlighted how the contributing factors to overweight/obesity vary between males and females. However, the study does have some limitations including that is cross‐sectional and is only able to present data at one point in time. As a result, we are not able to evaluate causality or take into account seasonal variations in nutritional status. Second, some of the dietary factors in the GDQS may be over or underrepresented due to self‐reporting bias. In addition, as discussed previously, the use of the GDQS does not take into account the amount or quality of the food eaten. Finally, as previously discussed, we had limited data on sexual maturation and the age of first period for females and were unable to take this into account in our analysis. That said, the results do provide useful insight into the burden of overweight/obesity in young adolescents living in Addis Ababa.

## 6 CONCLUSIONS

The study found that 8% of young adolescents in government schools in Addis Ababa, Ethiopia were overweight or obese. The proportion of females overweight or obese was significantly higher at 13%, compared with 2% of males. The contributing factors also differed between males and females. While none of the dietary variables were associated with overweight/obesity, both males and females had an inadequate diet and were at risk of poor health outcomes as a result. In addition, females were less physically active than males, which was associated with the prevalence of overweight/obesity. There is a need to improve the diet quality of young adolescents in Ethiopia and understand the reasons why females are less physically active to maintain low levels of overweight/obesity and to limit the risk of poor health outcomes.

## AUTHOR CONTRIBUTIONS

Amare W. Tadesse, Wafaie W. Fawzi, Roisin E. Drysdale, and Deepika Sharma designed the research and Amare W. Tadesse was responsible for conducting the research. Roisin E. Drysdale analyzed the data and wrote the first draft of the paper. Isabel Madzorera, Alemayehu Worku, Hanna Y. Berhane and Sachin Shinde provided feedback on the analysis and manuscript. All authors have read and approved the final manuscript.

## CONFLICT OF INTEREST

The authors declare no conflict of interest.

## Data Availability

Data described in the manuscript, code book, and analytic code will be made available upon request pending application and approval by the study team.
